# A study on the moral autonomy of adolescents—an empirical analysis based on the third time national moral survey

**DOI:** 10.3389/fpsyg.2026.1787524

**Published:** 2026-02-27

**Authors:** Yahui Chen, Jue Wang

**Affiliations:** School of Humanities, Southeast University, Nanjing, China

**Keywords:** adolescents, consciousness of moral norms, moral autonomy, moral persistence, moral worldview of adolescents, national moral survey, the unity of knowledge and action

## Abstract

**Introduction:**

Adolescents are energetic ethical existence of Chinese society. Moral autonomy among adolescents is a crucial factor influencing their development. This study focuses on the development of moral autonomy among adolescents and its influencing factors, with the goal of promoting adolescents’ well-being. The moral autonomy of adolescents is an essential factor in the development process of Chinese ethics and morality, which can be mainly examined from the four aspects of the moral worldview, the consciousness of moral norms, the unity of knowledge and action, and the moral persistence in conflict situations.

**Methods:**

A multi-source geospatial data-integrated, hierarchical and phased probability sampling method was employed. This approach introduced a high-precision half-grid system and utilized GPS/GIS-assisted address sampling, with GPS deployed for on-site positioning and verification. Data from the “Third National Moral Survey” were interpreted primarily through the “Structure–Cognition–Behavior” triadic framework. At the structural level, examined the influence of factors such as traditional culture and value systems on moral conceptions. At the cognitive level, explored internal psychological dimensions, including moral judgment and moral identity. At the behavioral level, observed actual gap between moral knowledge and moral actions.

**Innovation:**

In terms of innovation, this study constructs and empirically validates a comprehensive theoretical framework of “moral autonomy” among adolescents, supported by four empirically testable dimensions, thereby providing a new research direction for adolescent moral development. At the same time, by integrating scientific methods with a large-scale sample, and combining national probability sampling with validity verification, it offers an in-depth description of the developmental status of moral autonomy among adolescents.

**Results and discussion:**

According to the third time national moral survey data, the moral autonomy of adolescents has formed but not mature: In emerging fields of morality, a consensus on values has yet to be fully established; There exists a gap between moral action and moral knowledge; and moral cognition among adolescent groups remains incomplete and insufficiently thorough. Overall, this study will provide practical implications for youth-oriented pedagogical practices such as introducing dedicated case discussion curricula and developing “Digital Citizenship Ethics” curriculum system, and offer evidence-based recommendations for the formulation of related moral policies.

## 1 Introduction

As a group of critical national and social concern, Chinese adolescents represent a significant ethical entity and serve as a barometer for the future trajectory of moral development. The moral development of adolescents impacts the holistic progress of society (Chen L. Y. et al., 2025; Chen H. et al., 2025) and represents the future of social development, requiring attention from all sectors of society (Hamzagic et al., 2025). To better delineate the fundamental state of moral autonomy within this demographic, this study employs quantitative sociological research methods, conducting an empirical analysis based on data from the Third National Survey of Ethics and Morality undertaken by the Jiangsu Provincial Moral Development Think Tank.

The moral autonomy of citizens reflects the value ecology between civic morality and national ethics, serving as a crucial aspect of the ethical and moral development of a country and society (Zhao, 2025). Currently, adolescents face issues such as fragmented moral perspectives, ambiguous value standards, wavering beliefs, and resulting behavioral misconduct. Fostering moral autonomy among adolescents is instrumental in addressing these moral challenges (Yang and Zhao, 2024). Moral autonomy highlights the “power” of the moral subject in morality, reflecting a capacity—the ability to independently fulfill rightful missions and the subject’s autonomous arrangement of its own morality (Mancini, 2024). This power requires the bearing of both innate conditions and acquired foundations of the moral subject, which constitute the fundamental elements of moral autonomy; Moral autonomy requires the moral subject’s conscious identification with moral norms, which is the inherent essence of moral autonomy; Moral autonomy necessitates the unity of the moral subject’s moral cognition and moral action, which constitutes the core of moral autonomy; and Moral autonomy demands the moral subject’s ethical decision-making ability when confronted with major moral conflicts, for example, when facing difficult situations, if a moral individual lacks ethical decision-making ability, it may lead to negative moral outcomes such as failing to help others (Pummer, 2025). So, this is an important dimension of moral autonomy^1^. Therefore, the “moral autonomy of adolescents” discussed in this article is primarily examined from the moral worldview, self-awareness of moral norms, the unity of thought and action, and moral adherence in conflict situations. Through analyzing the moral autonomy of adolescents, this study aims to reflect on and explore the ethical strength of their moral autonomy, as well as whether their internal spiritual world and external social environment can provide strong impetus for the development of their moral autonomy, and its specific manifestations in situations of unity of thought and action and conflict.

The research questions of this manuscript are:

1. Is moral autonomy established among adolescents?
2. What issues exist regarding moral autonomy within adolescents?
3. What factors influence the moral autonomy of adolescents?

These questions, along with the responses to them, are presented in the main text, abstract, as well as the conclusion and discussion sections of the article.

### 1.1 Data source and study population

The survey data utilized in this study were jointly collected by the Jiangsu Provincial High-End Think Tank for Moral Development, the Publicity Department of the Jiangsu Provincial Committee of the Communist Party of China, and the Center for Chinese Social Research at Peking University. The target population consisted of Chinese citizens who had resided in mainland Chinese districts or counties for at least six consecutive months, excluding residents of Hong Kong, Macao, and Taiwan. The age of participants ranged from 12 to 20 years old.

### 1.2 Sampling design and method

A GPS/GIS-assisted address-based sampling method was employed to ensure spatial representativeness. The sampling unit was defined as half grid cells, with the DMSP/OLS nighttime light data used as a proxy for population density and regional scale. This approach enabled a stratified and geographically balanced selection of respondents across diverse socioeconomic and urban–rural contexts.

### 1.3. Validity and reliability

**Table 1:** 

Psychometric indicators of the four dimensions of moral autonomy (*N* = 8,488)					
Dimension	Number of Items	Cronbach‘s Alpha (α)	Average Variance Extracted (AVE)	Composite Reliability (CR)	Range of Standardized Factor Loadings (CFA)
Moral Worldview	5	0.82	0.51	0.83	0.58–0.79
Consciousness of Moral Norm	4	0.79	0.50	0.80	0.60–0.76
Unity of Knowledge and Action	3	0.76	0.52	0.78	0.62–0.80
Moral Persistence in Conflict	4	0.81	0.53	0.82	0.65–0.86

Overall Model Fit Indices *χ*^2^/df = 2.85, CFI = 0.94, TLI = 0.92, RMSEA = 0.047, SRMR = 0.038.

To ensure the quality of the survey data in this study, we conducted reliability and validity tests on the core dimensions of moral autonomy. First, confirmatory factor analysis (CFA) was employed to validate the hypothesized four dimensions of moral autonomy (namely, the consciousness of moral norms, the unity of knowledge and action, and the moral persistence in conflict situations). The analysis results indicated a good model fit (*χ*^2^/df = 2.85, CFI = 0.94, TLI = 0.92, RMSEA = 0.047, SRMR = 0.038). The standardized factor loadings of each item on its corresponding dimension ranged from 0.58 to 0.86, indicating good construct validity. Next, the internal consistency of each dimension was assessed by calculating Cronbach’s Alpha, yielding the following values: moral worldview (*α* = 0.82), the consciousness of moral norms (*α* = 0.79), unity of knowledge and action (α = 0.76), and moral persistence in conflict situations (α = 0.81). Cronbach’s Alpha for all dimensions exceeded the commonly used threshold of 0.70. The Average Variance Extracted (AVE) for each dimension exceeded 0.50, and Composite Reliability (CR) values were all above 0.78, indicating acceptable internal reliability. These psychometric indicators provide the necessary foundation of quality for subsequent data analysis and interpretation of results in this research.

### 1.4 Operationalization of constructs

In analyzing the data in this manuscript, abstract concepts were translated into measurable indicators. Using this table below as an example (though it does not cover all the examples used) helps to enhance the persuasiveness of the manuscript. This table lists the definitions and sample items for the four core dimensions of moral autonomy.

**Table 2:** 

Sample survey items for the four dimensions of moral autonomy			
Dimension	Conceptual definition	Sample survey Items	Response scale
Moral Worldview	An individual’s inherent natural disposition, along with their assessment of external moral conditions.	“The misfortune of others makes me uneasy” “I am satisfied with the current state of morality in our society.”	1 (Strongly Disagree) to 5 (Strongly Agree) 1 (Very satisfied) to 5 (Very unsatisfied)
Consciousness of Moral Norm	Conscious identification with and adherence to moral norms, such as traditional, mainstream, and modern values	“Virtues such as filial piety, humility, benevolence, and frugality are timeless and should never be abandoned.” “The core socialist values are beneficial for improving social conduct.”	1 (Strongly Disagree) to 5 (Strongly Agree)
Unity of Knowledge and Action	The consistency between an individual’s moral judgment/knowledge and their actual behavior in practice.	“I would never litter or spit in public.” (Behavioral self-report) “Willing to act and live as moral exemplar do”	1 (Never) to 5 (Always)/1 (Willing to follow) to 5 (Resolutely not do)
Moral Persistence in Conflict	Uphold moral principles when faced with ethical dilemmas, interpersonal conflicts, or pressures involving significant personal interest.	“If I had a major conflict of interest with a family member, I would choose to communicate directly and be forgiving.” “In a conflict with a business partner, I would resort to legal action.”	1 (Strongly Disagree) to 5 (Strongly Agree) The results are presented in proportion

### 1.5 Data collection procedure

The survey was conducted through face-to-face interviews using a structured questionnaire. Trained interviewers administered the instrument, which covered topics related to values, ethics, social attitudes, and demographic characteristics. Quality control measures included interviewer training, field supervision, and logical consistency checks during data entry.

### 1.6 Sample characteristics

The final sample comprised 8,488 valid responses distributed across 76 district- or county-level administrative units. The sample demonstrated strong representativeness in terms of geographic distribution, urban–rural composition, and key socioeconomic indicators, thereby providing a reliable basis for generalized inferences regarding the target population.

## 2 The moral worldview of adolescents

Moral autonomy is the extent to which a moral agent “gathers their spirit and becomes their own master” in ethical matters. The internal state of a moral worldview serves as a fundamental indicator of a moral agent’s moral autonomy. The moral worldview encompasses the relationship between the moral agent and subjective nature as well as objective nature. Subjective nature refers to the individual’s innate natural disposition, while objective nature primarily denotes the external, objective order (Fan, 2017). The moral worldview reflects the inherent moral conditions and acquired moral environment of the moral agent. Studying the moral worldview of adolescents forms the foundation for researching their moral autonomy. Research indicates that the moral worldview of adolescents possesses a sound innate foundation. Adolescents critically evaluate the external moral environment, exhibiting a degree of ethical impulse and expectation. Conscience plays a crucial driving role in connecting their subjective and objective nature.

### 2.1 The subjective nature of adolescents

Adolescents are increasingly viewing society as their ethical laboratory, and “‘everything is permitted’ has become the distinctive ‘moral temperament’ of the master of this ‘ethical laboratory’” (Fan, 2010). It is precisely for this reason that the subjective natural state of the adolescent group appears particularly important. Naturalist educator Rousseau firmly defended humanity’s natural disposition, believing that it represents the original tendencies and innate abilities of human beings. He argued that humans are inherently good and pure by nature, and that education should be conducted in accordance with these fundamental human traits (Rousseau, 1962). Laozi’s view of nature shares similarities with Rousseau’s regarding human natural disposition, as both advocate for a philosophical state of embracing simplicity and returning to one’s original, unadorned nature. Therefore, natural instincts, as the innate abilities and conditions of human beings, serve as the inherent soil for all social norms to take root, and also mark the beginning of moral existence for adolescents as they face the external world. In modern society, how does the natural disposition of adolescents manifest itself? Can subjective nature provide an innate foundation for the morality of the adolescents?

This survey assessed the natural disposition of adolescents through several subjective questions. 96% of adolescents demonstrated varying degrees of concern for those less fortunate than themselves, while 97.7% expressed varying levels of sympathy for the difficulties faced by others. Additionally, 96.5% of adolescents make varying degrees of effort to consider issues from multiple perspectives before making decisions, while 94.2% experience some level of desire to protect those who are being taken advantage. Furthermore, 84.9% feel varying degrees of unease in response to the misfortunes of others. These findings indicate that adolescents possess positive innate qualities such as love, compassion, empathy, and a sense of justice within their subjective nature, reflecting an optimistic and upward state of their natural disposition. Adolescents, while expressing their natural disposition, also demonstrates the ability to consider moral judgments of right and wrong, such as recognizing that “taking advantage of others” is wrong. In the context of moral propositions, human nature is the sum of emotions and desires, serving as the primary foundation for morality (Li, 2020). Therefore, the natural disposition of adolescents can constitute the innate foundation for moral existence.

### 2.2 The objective nature of adolescents

Adolescents possess their own moral positioning and ethical expectations. According to Confucian philosophy, humans are inherently moral beings, endowed with certain innate ethical demands (Yang, 2018). According to survey data, the objective moral situation can generally meet the moral expectations of adolescents, but there is a gap between the moral requirements of adolescents themselves and the actual external moral situation. 8.7% of adolescents are very satisfied with the overall moral situation in our country, 62.3% of them choose the option of “relatively satisfied,” and 29% of them are “not very satisfied” and “very dissatisfied” with the overall moral situation in our country. Regarding the survey item “What is your overall satisfaction with the current state of interpersonal relationships in Chinese society?,” the results are largely consistent with the former findings. Despite the gap between internal moral demands and external reality, the adolescents remains optimistic about the future development of morality in China, with 74.3% of adolescents believing that the moral condition of Chinese society will continue to improve moving forward. Only 6.7% adolescents gave a negative evaluation of China’s future moral situation, indicating that although the majority of adolescents (29%) who are “not very satisfied” and “very dissatisfied” with China’s overall moral situation experience the imperfections of the moral situation, they still have positive expectations for the future moral situation. Since there is a gap between the moral demands of adolescents themselves and the actual external moral conditions, in what specific aspects is this gap reflected? The survey shows that “inability to eradicate corruption” was chosen by 38.1% of adolescents, ranking first; “deterioration of the ecological environment” was chosen by 31.5%, ranking second; and “strained interpersonal relationships” was chosen by 22%, ranking third. The focus lies in three areas: politics, ecology, and society. Corruption is morally reprehensible behavior (Miller, 2018); nature embodies the essence of humanity (Cao, 2010); and the ethical state of interpersonal relationships is a significant reflection of the overall functioning of society (Harris, 2011). “Eradicating corruption,” “promoting harmony with nature,” and “fostering virtuous social interactions” are the key moral expectations of adolescents toward politics, society, and nature. Since there is a gap between moral expectations and the actual external moral conditions, where do adolescents place their moral aspirations and pursuits? The answer lies in traditional Chinese culture. Traditional Chinese culture is by no means a relic of the past; rather, it is a continuous spiritual flow that extends from history into the present and onward toward the future. It connects the past, present, and future, serving as the spiritual lifeblood of the Chinese people (Zhu, 2010). The ethical values of traditional society are admired and pursued by more than half of adolescents, with benevolence, righteousness, propriety, wisdom, and credibility still holding unique cultural appeal in contemporary times. At the same time, there exists a gap between the internal moral demands of adolescents and the current state of objective nature, reflecting the autonomy and self-awareness of morality within this group. Although it is not yet possible to intuitively perceive the awareness of adolescents toward their own ethical essence, their moral expectations for the future to some extent align with the characteristic of moral agents being “self-determining.”

### 2.3 Conscience

**Figure 1:**
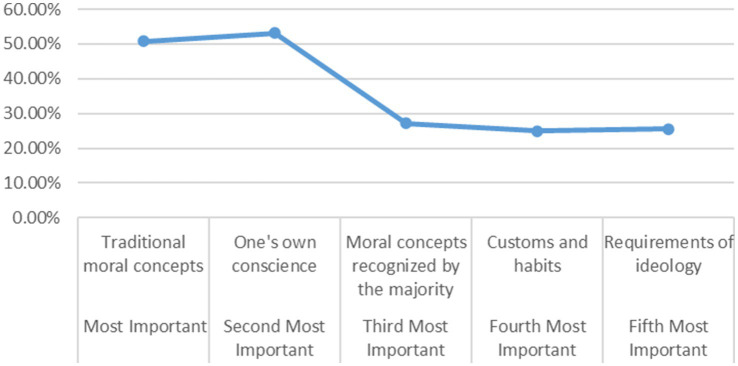
Ranking of “criteria for determining whether a behavior aligns with ethics or morality”.

In the field of psychology, conscience is a highly significant concept. The psychology dictionary defines conscience as internalized moral principles that provide guidance for behavior (Reber, 1996). It represents a commitment to and awareness of moral obligations, closely intertwined with our consciousness, empathy and emotions (León, 2006). Conscience manifests itself within social relationships and moral affect, subtly influencing an individual’s behavioral patterns (Apressyan, 2019). Conscience is the subjective self-awareness’s firm judgment of its own internal rights and obligations, representing the unity of subjective cognition and being-in-and-for-itself. As conscience, which exists in the relationship of the self with itself, it abolishes external limitations and frameworks, fully becomes its own essence, and transcends the constraints of particular purposes to aspire to goodness in and for itself (Hegel, 2017). It is an internally proactive goodness in thought and emotion, directed toward the conscious benevolence within the human heart. It pertains to the state of human consciousness and represents a manifestation of value judgment (Xie, 2018). Thus, conscience is an ability of the self to coexist with itself—a spontaneous, pure form of goodness that unifies the internal and external worlds, serving as a crucial foundation for moral behavior. To examine the role of conscience in the moral judgment of the adolescents, this questionnaire used a ranking question to survey their choices regarding the basis for judging “whether a certain behavior conforms to ethics or morality.” The survey results are presented in Figure 1.

Traditional Chinese culture boasts a long history, and possesses a unique cultural tradition and a rational value core, making it highly worthy of preservation and transmission (Wang and Dong, 2016). With its profound historical roots, traditional culture serves as a vital carrier for cultural inheritance among the adolescent group. Consequently, traditional moral concepts naturally become the nurturing ground for the moral development of this generation. “Traditional moral concepts” were ranked by adolescents as the “most important,” while “one’s own conscience” was ranked as the “second most important.” The remaining options showed little significant difference in terms of importance. Therefore, traditional moral concepts are the primary factor influencing adolescents’ ethical and moral judgments. When selecting the “second most important” option, with the absence of “traditional moral concepts” as an influence, most adolescents chose “one’s own conscience.” The selection and affirmation of one’s own conscience indicate that adolescents possess internal criteria for self-evaluation. The subjective nature of conscience and its internal demands for moral behavior play a crucial role in bridging subjective and objective nature. This conscious internal goodness plays a significant role in driving the process through which adolescents develop into moral agents.

The analysis of adolescents’ subjective nature, objective nature, and conscience in this section has undergone rigorous validation. The standardized factor loadings for each item under the “moral worldview” dimension range from 0.58 to 0.79. Furthermore, *α* = 0.83, indicating that the measurement tool can reliably and consistently capture the subjective and objective states of adolescents.

## 3 The consciousness of moral norms

As social beings, humans require the guidance of moral norms to maximize the collective social welfare. The binding force of moral norms stems from the autonomous will of the acting agent. At the same time, we grow up within an intergenerational moral environment, where the content of moral norms evolves across generations, reflecting a coexistence of traditional and contemporary characteristics (Gan, 2011). There is no doubt that traditional moral concepts form the core of moral norms. Mainstream values represent moral norms with clear stipulations in political, economic, cultural, and social dimensions, while widely endorsed yet unwritten modern value consensus also constitutes a vital part of moral norms. Therefore, the level of consciousness toward moral norms can be examined from perspectives such as outstanding traditional culture, mainstream values, and modern value consensus. Studying the consciousness of moral norms among adolescents is essential to understanding the intrinsic meaning of moral autonomy within this group. Research indicates that adolescents can consciously identify with moral norms, and this identification exhibits a characteristic of a fusion of tradition and modernity. Traditional moral norms remain the most important norms that adolescents adhere to.

### 3.1 The primary moral norm: outstanding traditional culture

The consciousness of moral norms among adolescents should first consider their awareness of traditional moral norms, as these norms have been present since birth and will continue to influence their lives throughout. Culture is fluid and forward-moving, with individuals serving as its carriers. If culture is not embraced, it cannot be preserved (Fei, 2004). Adolescents are key participants in traditional education and exist within an ever-evolving generational cycle. The influence of traditional culture they experience now will, as they mature into responsible individuals, inevitably shape their own offspring, thereby laying the foundation for the cultural construction of the next generation of youth (Xu, 2019). Therefore, examining the current acceptance of outstanding traditional culture is a primary aspect of assessing the moral norm consciousness among the adolescents.

Chinese society is advancing toward the modern world, and while lifestyles and ideologies have undergone dramatic changes compared to the past, the outstanding traditional concepts remain a major spiritual pillar for the Chinese people. For an extended period in history, Confucianism served as the orthodox ideology of traditional Chinese society, gradually evolving into a widely accepted cultural philosophy. It forms the mainstream of traditional Chinese culture, with its profound yet concise teachings and alignment with human nature being the source of its enduring vitality. The survey indicates that among the fine traditions such as “filial piety, humility, benevolence, and frugality,” 75.5% of adolescents believe that “these good traditions must never be abandoned.” Filial piety, humility, benevolence, and frugality are precisely the core values of Confucianism. “A gentleman focuses on the fundamentals; when the fundamentals are established, the way is born. Filial piety and fraternity are the foundation of benevolence (The Analects: Xue Er).” “In the application of rites, harmony is most valued. The way of the ancient kings was beautiful in this respect, in matters both small and great, they followed this principle. (The Analects: Xue Er)” “If a man lacks benevolence, what can he have to do with ritual? If a man lacks benevolence, what can he have to do with music (The Analects: Ba Yi)?” “A respectful person does not insult others; a frugal person does not seize from others (Mencius · Li Lou I).” Filial piety is regarded as a natural and fundamental principle in traditional Chinese culture, while benevolence, humility, and frugality serve as the foundational virtues for personal cultivation, family harmony, state governance, and the pursuit of universal peace in Confucian philosophy. According to the survey, among Chinese adolescents, 9.5% believe that these fine traditions are “optional,” 6% consider them “outdated and unnecessary,” and 8.9% think “some should be kept while others discarded.” In contrast, 82.6% of adolescents believe that it is “highly necessary to promote traditional education across society, particularly among young people, as one must never forget one’s roots at any time.” This indicates that while there may be certain challenges in integrating outstanding traditional culture with modern society due to changes in the environment, these issues do not diminish its dominant role. Therefore, traditional Chinese culture continues to serve as the primary moral compass for adolescents, with Confucian thought remaining a core component of this cultural heritage.

### 3.2 Mainstream values

Values refer to the conscious activities or processes of individuals or groups, representing the subject’s value assessments and judgments regarding specific domains and matters. These reflect the subject’s value goals and ideal pursuits (Cen, 2007). Mainstream values are the evaluative criteria and value benchmarks recognized and accepted by the broad masses of the people. In contemporary China, mainstream values refer to the reflection of the dominant social economy and the value concepts widely accepted and endorsed by members of society. They possess universal discursive power (Wu, 2016), with core socialist values serving as the central expression of China’s mainstream values (Chen, 2006). The degree of identification with core values reflects the extent to which adolescents accept prevailing social norms, and serves as an important indicator of their moral consciousness and self-discipline. Research indicates that core socialist values enjoy a relatively high level of recognition among adolescents, and the ranking of their acceptance of these values exhibits distinct preference characteristics. The focus of adolescents is primarily centered on the construction of civic morality, while also acknowledging the leading role of modern civilization in guiding this endeavor.

Prosperity, democracy, civility, harmony, freedom, equality, justice, the rule of law, patriotism, dedication, integrity, and friendliness constitute the core values of China. They form the essence of the core socialist value system and reflect the emotional identification of the Chinese people with this system. These values represent a distillation of principles at the national, societal, and individual levels. 86.3% of adolescents believe that the Core Socialist Values contribute positively to improving social conduct, asserting that everyone should adhere to these principles in their actions and decisions, as they are closely linked to both work and daily life. This indicates that the Core Socialist Values are consciously accepted and recognized by the vast majority of adolescents, with their significance and value also being voluntarily affirmed and endorsed by most of the adolescents.

In this survey, adolescents were asked to rank the top four values they consider most important. The results show that Civility (75.8%), Integrity (68.5%), Patriotism (56.9%), and Friendliness (47.3%) were ranked first, second, third, and fourth, respectively, in their choices. “Civilization” is one of the core values at the national level, representing one of the highest tiers of values within the framework. Integrity, patriotism, and friendliness, on the other hand, pertain to civic ethical norms and fall under the category of individual-level values. It is evident that adolescents demonstrate a comprehensive recognition of the civic moral norms within the core socialist values. Among these, “Civility”—a value at the highest national level—ranks first, resonating deeply with other civic ethical norms such as “Integrity,” “Patriotism,” and “Friendliness.” This indicates that civic morality constitutes a central focus for young people, with “Civility” occupying a guiding and overarching position in their value system. Civility is the outcome of humanity’s endeavors to fulfill fundamental needs and achieve all-round development. It encompasses both the material and spiritual achievements we have attained through overcoming challenges, reflecting humanity’s enduring pursuit of truth, goodness, and beauty (Chen, 2006). From the “Five Stresses and Four Beautifications” to the Core Socialist Values, the concept of “stressing civilization” has taken deep root among the people. This demonstrates that the advocacy of “promoting civility and fostering new social practices” has yielded tangible results. The adolescent demographic represents an evolving ethical community whose values and outlook on life are continually maturing and refining. Their emphasis on integrity, patriotism, and friendliness serves as a concentrated reflection of their internalized moral demands. The relative lack of emphasis on societal-level core values among this group is closely related to the developmental characteristics of adolescents.

### 3.3 Modern value consensus

Value consensus refers to the relatively consistent common understanding and perspective that members of society reach regarding specific value concepts in social life through social interaction and practice. It belongs to the realm of ideas that represent the long-term interests of the vast majority of people. Value consensus represents both an enhancement and a complement to diverse values, heralding the potential for individuals to awaken to a sense of coexistence with others (Wang and Huang, 2012). Any value consensus is historically situated, serving as a theoretical reflection of its time and social practice (Chen, 2009). Modern value consensus permeates all aspects of social and moral life. Research indicates that contemporary society exhibits a strong awareness of value consensus, having largely fostered a positive atmosphere for its development among adolescents. However, certain divergences persist within adolescents regarding emerging dimensions of modern value consensus, such as cyber ethics.

**Figure 2:**
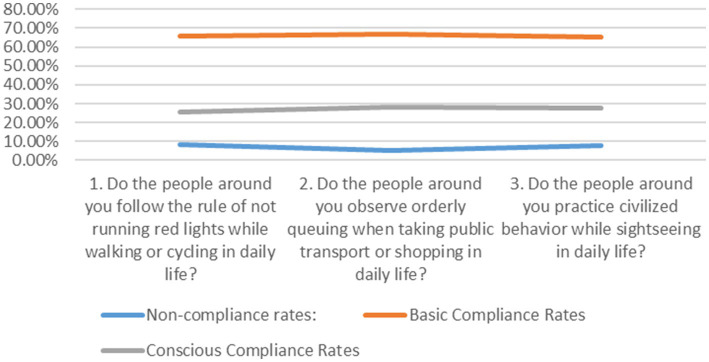
Compliance with public order.

This survey assessed whether adolescents are immersed in a positive atmosphere of value consensus by asking them three sets of questions. The results are presented in Figure 2.

In the moral environment surrounding adolescents, over 90% of social members demonstrate basic or conscious compliance with public order. This reflects a high degree of social consensus and voluntary adherence to shared values, which in turn exerts a positive and proactive influence on fostering value consensus awareness among adolescents. Under a positive atmosphere of social consensus, adolescents ranked the virtues they consider most essential and important in today’s society as follows: the top four are love (32.6%), responsibility (16.7%), integrity (15.9%), and filial piety (13.2%). The virtues most valued by adolescents in contemporary society show some divergence from the core socialist values, yet remain aligned with the essence of traditional culture. This indicates that outstanding traditional culture has become the ideological core of the younger generation. Mainstream values and value consensus serve as important moral norms for adolescents in modern society, complementing and reinforcing each other.

In emerging areas of social morals, such as internet morals, 79% of adolescents agree that “although the internet is a virtual space, it is still subject to the moral norms of real life.” Additionally, 73.2% of adolescents agree that “doxing infringes on personal privacy should be eliminated.” However, regarding internet morals, the level of consensus on values among adolescents is relatively low. In virtual space, a new moral domain, there is a divergence among adolescents in their understanding of whether “cyberspace constitutes a moral space.” This indicates that, with the expansion of moral domains in modern society, the formation of moral consensus in emerging fields faces distinct challenges from traditional moral constructs. At the same time, due to increased societal openness and the arrival of an era of value pluralism, the views of social groups—including teenagers—on specific domains or moral issues are increasingly diverse. The divergence observed among adolescents regarding internet morals precisely reflects the characteristics of our time.

This section focuses on the consciousness of moral norms, exploring three aspects among adolescents: outstanding traditional culture, mainstream values, and modern value consensus. Cronbach’s Alpha is 0.79. In the confirmatory factor analysis, the factor loadings of the items ranged from 0.60 to 0.76, accurately reflecting the consciousness of moral norms among the adolescents.

## 4 The unity of knowledge and action

From a literal perspective, the unity of knowledge and action means that what one knows and what one does are consistent and not contradictory. Wang Yangming, a master of the School of Mind, first proposed the doctrine of the unity of knowledge and action during his lectures at Wenchang Academy in Guiyang. “Nowadays, everyone knows that one should be filial to parents and respectful to elder brothers, yet many fail to practice filial piety and fraternal duty. This shows that knowledge and action are clearly two separate matters” is not evidence that “knowledge” and “action” are separated. This precisely indicates that “it is already severed by selfish desires and is no longer the true essence of knowing and doing.” “There has never been one who knows but does not act. To know without acting is simply not yet to know (“A Record of Practicing Unity of Knowledge and Action, Part One: Instructions for Practical Living, Volume One”).” For Wang Yangming, “the stirring of a single thought” constitutes “action,” and the essence of “knowledge and action” lies in the subject’s effort to eliminate selfish desires. However, the “stirring of a single thought” alone is insufficient to drive tangible ethical practices in society, nor can moral conduct remain confined solely within subjective consciousness. The essence of moral lies not only in the consciousness of knowledge but also in the self-discipline of action, which is the unity of knowledge and action (Fan, 2017). Therefore, the unity of knowledge and action primarily examines whether the moral knowledge and moral behavior of adolescents are consistent. Investigating the state of this unity among adolescents serves as a core component in assessing their moral autonomy. The survey indicates that while adolescents possess a strong understanding of morals, they are often unable to translate this moral awareness into practical actions at the same level due to the influence and constraints of various internal and external factors.

Over 90% of adolescents consider behaviors such as spitting in public, cutting in line, talking loudly on the phone on busses or subways, making excessive noise in restaurants, and sleeping on chairs or sofas in public places to be unmoral. However, only about 70% of adolescents report that they have never engaged in the behaviors mentioned above, while approximately 30% admit to doing so frequently or occasionally. On the question, “Have you heard stories about moral role models? Would you be willing to act and live as they do?” 56.2% of adolescents responded, “I know some, they are remarkable, and I should strive to learn from them”; 28.3% said, “I know some, I deeply admire them, but I do not think I can emulate them”; and 4.7% answered, “I know some, but I feel it’s somewhat not worth it to act the way they do.” Therefore, while adolescents already possess a fundamental understanding of morals, their moral conduct in real-life situations often falls short of aligning with their moral awareness. There exists a certain gap between adolescents’ moral behavior and their moral cognition.

**Figure 3:**
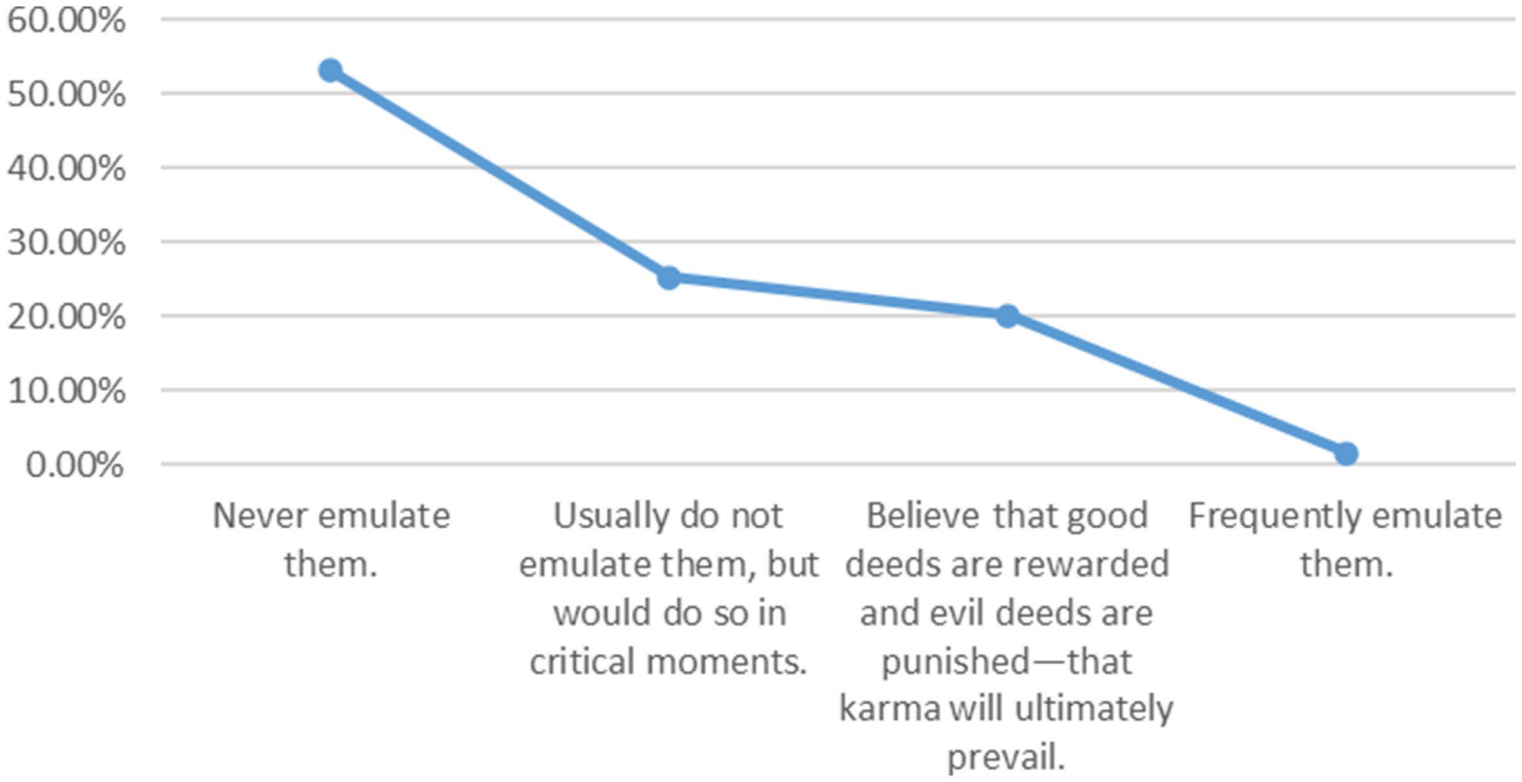
Imitation of unethical behaviors.

Regarding the imitation of unethical behaviors, only 1.5% of adolescents reported frequently imitating such behaviors, while 53.1% stated that they never imitate unethical behaviors. The remaining adolescents chose “usually do not imitate, but would do so in critical moments” and “believe that good is rewarded and evil is punished, and that justice will ultimately prevail,” accounting for 25.3 and 20.1%, respectively. The concept of karma is a significant element in human ethical thought, embodying a reverence for the “punishment of evil” (Sun, 2014). While it is not possible to directly determine the attitude of the 20.1% of adolescents who believe in karma toward imitating unethical behaviors, it can generally be inferred that their stance falls within the range of “not imitating” to “occasionally imitating.” The fact that 25.3% of adolescents would resort to imitating unethical behaviors in critical moments precisely illustrates that, despite possessing moral knowledge and judgment, some young people still choose unethical actions at pivotal times when they are called upon to practice ethical conduct. The imitation of unmoral behaviors among adolescents is influenced by external factors such as real-world conditions and cultural norms, while also reflecting, to some extent, their concern for the ethical consequences of such actions (Figure 3).

Among the responses from adolescents regarding the question, “Knowing something is moral but not taking action—what is the biggest obstacle to acting?” the top four reasons cited were: “limited personal ability, having the will but lacking the means,” “taking action is unlikely to achieve the desired outcome,” “taking action would harm my own interests,” and “since no one else is doing it, why should I bother?” This indicates that, despite possessing fundamental moral knowledge, adolescents face limitations—both internal and external—when making choices about moral actions. Factors such as moral competence and self-interest act as internal barriers to their moral behavior, while moral outcomes and the ethical environment serve as external obstacles. While adolescents have not yet fully achieved the unity of knowledge and action, they already possess a basic understanding of morals. The morality of their behavior is influenced by both internal and external factors, and the ethical consequences of their actions are a matter of concern to this group.

This section reveals a gap between the moral knowledge and action of adolescents. The measurement of this gap consistent with the psychometric indicators of the four dimensions of moral autonomy (*N* = 8,488). All psychometric indicators meet the required standards: Cronbach’s Alpha is 0.76, and confirmatory factor analysis shows that all items are effectively loaded onto a single factor, with loadings ranging from 0.62 to 0.80. This indicates that the observed “gap” such as the high level of moral knowledge (>90%) and the relatively lower level of action compliance (approximately 70%), as well as the complex factors influencing moral behavior, are presented within a valid and reliable measurement framework.

## 5 Moral persistence in conflict situations

Moral conflict is an objectively existing phenomenon in human society. One of its key manifestations is the contradictory state in which individuals find themselves when making behavioral choices within specific moral contexts. Conflicts within moral contexts often require individuals to weigh interests, primarily reflected in the clash of moral values and the contradictions between specific moral choices and evaluations (Wang, 2016). Moral conflict exists in every aspect of life, and it is within such conflicting situations that moral persistence is most clearly demonstrated. Studying the moral choices of adolescents in situations of moral conflict is a crucial dimension for understanding their moral autonomy. Research indicates that in moral conflict situations, adolescents’ moral persistence reflects a strong emphasis on ethical relationships, such as those with family, friends, and colleagues, and their approaches to resolution often carry significant ethical considerations. In contrast, when dealing with commercial partners and similar relationships, they tend to rely more on modern approaches to resolve issues, with their methods clearly reflecting the characteristics of contemporary interpersonal interactions. Therefore, adolescents employ two distinct ethical systems for handling moral persistence in conflict situations.

To examine the moral persistence of adolescents in conflict situations, this survey uses four hypothetical questions to explore their ethical responses when facing major conflicts of interest with family, friends, colleagues, and business partners. As the ethical closeness diminishes across the four social relationships—family, friends, colleagues, and business partners—the proportion of adolescents who choose to “resort to legal action, file a lawsuit” gradually increases when major conflicts of interest arise. The highest percentage opting for litigation is observed in conflicts with business partners, significantly exceeding the proportions for family, friends, and colleagues. The willingness to “communicate directly with the other party but exercise reason and restraint, knowing when to stop” and to “exercise tolerance and forbearance whenever possible” gradually decreases from family and friends to colleagues and business partners. In all ethical relationships, family ethics are the most fundamental and natural. The ethical order of traditional Chinese society also begins with the family and extends outward—from family to friends, and then expands to the broader society (Wang and Yu, 2016). Friendship is a close relationship formed between like-minded individuals, characterized by mutual assistance and goodwill. It embodies both personal and social dimensions (Jie, 2016) and is an integral part of traditional ethical frameworks. In contrast, colleagues and business partners represent modern interest-based relationships, where the connections with them are highly social in nature. Therefore, when faced with significant conflicts of interest, individuals are more inclined to exercise tolerance and restraint toward family and friends and are less willing to resort to legal action or litigation. In dealing with colleagues and business partners, however, the approach tends to be more formal.

Only 16.3% of adolescents choose to “seek mediation through a third party to minimize damage to the relationship” when facing major conflicts of interest with family members, making this the lowest proportion among all options. In contrast, 38.4% of adolescents are willing to resolve conflicts with colleagues through third party mediation, representing the highest proportion for this approach. It is evident that for conflicts of interest within the family, the vast majority of adolescents are unwilling to involve a third party, i.e., outsiders. Therefore, the handling of ethical relationships within the family tends to be characterized by a high degree of privacy and insularity. Career development is a crucial aspect of an individual’s social life, and relationships with colleagues represent an important form of cooperation. Therefore, when it comes to colleague relationships, there is a greater willingness to involve third parties to avoid damaging harmony among coworkers. Apart from directly approaching the other party to resolve issues, adolescents also tend to rely on third party mediation to address conflicts of interest between friends. This further reflects the social dimension inherent in friendship. Therefore, third parties serve as crucial mediators in resolving conflicts of interest between friends and colleagues. The emergence of two distinct ethical attitudes among adolescents when handling major conflicts of interest reflects their corresponding ethical discernment in managing relationships across different domains.

**Figure 4:**
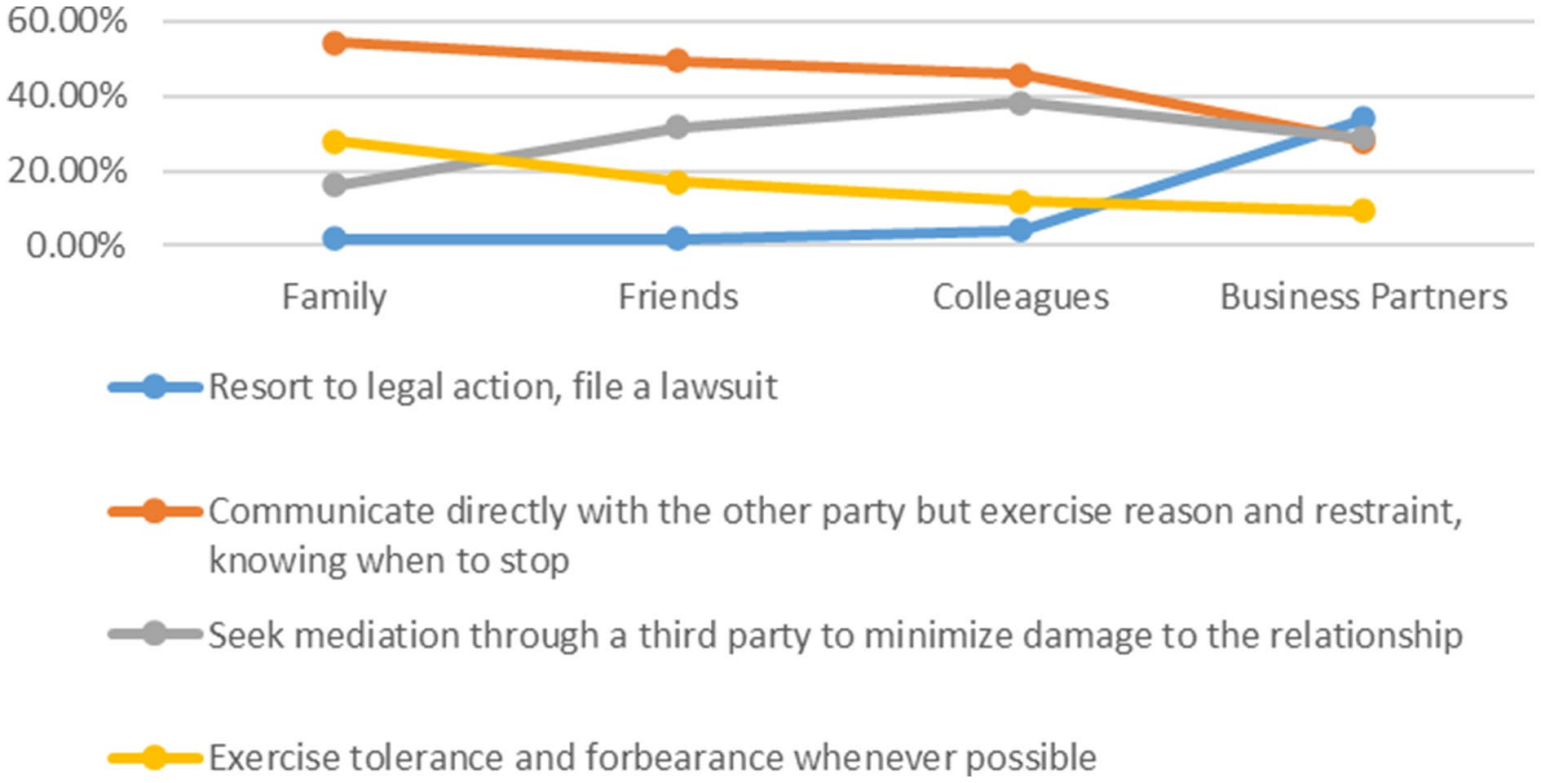
Ethical choices in situations of major conflicting interests.

This section’s analysis of the decision-making patterns of adolescents in conflicts across different ethical relationships relies on the psychometric indicators of the four dimensions of moral autonomy (*N* = 8,488). The scale demonstrates excellent psychometric properties: Cronbach’s Alpha is as high as 0.81, and confirmatory factor analysis reveals high factor loadings for the situational items (ranging from 0.65 to 0.86). Therefore, the gradient shift in ethical responses from “family” to “business partners” presented in Figure 4—where emotional and relational approaches decrease as relationships become more distant, while instrumental and rule-based solutions correspondingly increase—exhibits high measurement stability and structural validity.

## 6 Conclusion and discussion

First, a positive innate nature (Diener, 1984) and a sound social-moral environment provide a favorable innate foundation (Pu et al., 2025) and nurturing soil for the formation and development of moral autonomy in adolescents. Conscience supports the inner goodness of adolescents (Mazibayeva, 2023) and fosters moral consciousness within adolescents. Adolescents are capable of viewing the surrounding moral world with a critical eye (Yan et al., 2025) and expressing concern and worry about social issues (such as corruption and environmental problems). There is a gap between the actual moral conditions in society and the traditional moral standards recognized by adolescents (Yi, 2025), which leads this group to hold further moral expectations for social development (Hardy et al., 2011).

Second, adolescents exhibit a good sense of moral awareness (Zhang, 2017), and Chinese traditional culture—particularly Confucian culture—holds a high degree of moral identity among adolescents (Chen and Qin, 2024). There is a lack of societal-level attention to adolescents’ identification with the core socialist values (Li, 2023). While adolescents are in an environment conducive to value consensus, they exhibit some divergence in their understanding of moral norms within emerging ethical domains (Flores and James, 2013). As a result, a full consensus on values in these new moral fields has yet to be achieved.

Third, adolescents possess a good understanding of moral knowledge (Hardy et al., 2014) and maintain a certain reverence for the concept of “karma” (Tang and Guo, 2019). However, there is a lack of sufficient motivational support for their moral behavior within this group, leading to a gap between moral action and moral knowledge^2^ (Darnell et al., 2019). Additionally, moral disengagement behaviors exist among adolescents (e.g., expressions such as “it’s thankless work” appearing in questionnaires) (Guo et al., 2023). Moral competence, self-interest, moral outcomes, and the specific moral environment serve as internal and external factors contributing to the phenomenon of “knowing but not doing” among adolescents. These factors can easily lead to cognitive dissonance in their moral understanding (Kaswa, 2025).

Fourth, in situations of moral conflict, family ethical relationships remain the core of adolescents’ ethical framework (Fan, 2020). Adolescents tend to be more tolerant toward family members and friends (Du, 2025), while showing lower tolerance toward business partners and colleagues, and are more likely to resort to legal measures. Adolescents exhibit a certain degree of ambivalence in their approach to colleague relationships: on one hand, they desire harmonious interactions (Moore et al., 2022), while on the other, they tend to opt for legal avenues when conflicts arise (Chen L. Y. et al., 2025; Chen H. et al., 2025). This, to some extent, reflects the complexity of contemporary interpersonal relationships.

### 6.1 Study limitations

While this study strives for rigor in terms of data scale and research design, several noteworthy limitations remain. Future research could build upon these to achieve further depth:

(1) Methodological Limitations: Social Desirability Bias. Although standardized scales were employed and subjected to rigorous reliability and validity tests, data collection relied primarily on self-reporting. When responding to sensitive questions involving moral judgment and social norms, adolescent participants may have exhibited social desirability bias—that is, a tendency to provide answers that align with mainstream social values. This may have influenced, to some extent, the depth of authenticity and the expression of nuanced differences in the data.

(2) Limitations in Sample Representativeness. The sampling for this study was concentrated in 76 districts and counties within mainland China. Although the sample demonstrated good internal representativeness in terms of urban–rural distribution and economic development levels, it did not include adolescents from Hong Kong, Macao, Taiwan, or overseas Chinese communities. The manifestations and influencing factors of moral autonomy may differ systematically among adolescents under different social systems, educational environments, and cultural contexts. Therefore, caution is advised when generalizing the findings of this study to broader Chinese cultural spheres or employing them for cross-cultural comparisons. The generalizability of the results warrants further testing across wider geographic and cultural ranges.

(3) Research Design Limitations. This study is based on cross-sectional survey data. While it clearly depicts the current state of adolescents’ moral autonomy across multiple dimensions and identifies relevant factors, it cannot trace dynamic developmental pathways over time. For example, whether the gap between moral knowledge and moral behavior represents a stage in individual development or is the result of specific environmental influences cannot be determined from the current data alone. Future research could adopt longitudinal designs or intervention experiments to further uncover the internal mechanisms and key turning points in the development of moral autonomy.

(4) Limitations in Controlling for Environmental Variables. While this study examines the influence of the social moral environment on adolescents, the data were collected within a relatively concentrated timeframe. This limits the ability to adequately account for potential phase-specific effects of macro-social changes, such as major public events, reforms in educational policy, or the evolution of new media ecosystems. Subsequent research could attempt to span longer timeframes, combining longitudinal data with contextual analysis, to more comprehensively reveal the relationship between moral development and the broader socio-temporal context.

### 6.2 Practical implications

The findings of this study offer clear practical implications for adolescent moral education:

On the basis of existing traditional culture and core values education, it is recommended to introduce dedicated case discussion curricula focused on moral dilemmas (McLeod-Sordjan, 2014). By leveraging adolescents’ intrinsic identification with “conscience,” educators can design emotional experience and reflective training activities—such as role-playing and moral narrative writing—to strengthen the connection between moral emotion and self-awareness (Liu et al., 2024), thereby reducing moral disengagement.

Develop a dedicated “Digital Citizenship Ethics” curriculum system (Mirra et al., 2022). In response to the research findings that indicate adolescents have low consensus and ambiguous normative understanding in the field of online ethics, it is recommended to integrate digital ethics education into compulsory modules. The curriculum should focus on core issues such as the social consequences of online behavior and the ethics of responsibility in virtual communities, aiming to help adolescents establish ethical consensus in this domain.

Establish an ethical support system. By offering family ethics communication workshops (Boechat, 2022), setting up campus ethics counseling centers, and establishing annual moral practice bases, we can provide adolescents with ethical support for their moral development. This system will enable adolescents to experience the practical forms of moral norms through diverse social roles (Gawronski and Brannon, 2020).

## Data Availability

The original contributions presented in the study are included in the article/supplementary material, further inquiries can be directed to the corresponding author.

## Footnotes

1 The four dimensions of moral autonomy are derived from the academic research report titled *China Ethical and Moral Development Report: National Volume (Two Volumes)*, co-authored by Fan Heping (pen name Fan Hao) and Wang Jue (my supervisor), and published by China Social Sciences Press in 2022. This report includes the China Ethical and Moral Development Assessment, which was jointly developed by the Ethics Research Team of Southeast University, the Publicity Department of the Jiangsu Provincial Committee of the Communist Party of China, and Peking University. In this assessment, moral worldview, the consciousness of moral norms, the unity of knowledge and action, and moral persistence in conflict situations serve as key indicators for measuring moral autonomy. The rationale is as follows:

1. The formation of morality and moral subjects is marked by what is termed the “moral worldview,” whose level of conscious development is a fundamental indicator of citizens’ moral maturity.
2. The core of morality consists of norms, and the moral autonomy of an individual is first manifested through a conscious adherence to moral norms.
3. The essence of morality lies not only in the acquisition of knowledge but also in self-regulated behavior, so, “unity of knowledge and action” is needed.
4. Moral autonomy is not merely about action but also about persistence and steadfastness, making conflict situations a more reliable context for assessing the level of moral development.

My foundational knowledge in ethics was imparted by Professor Wang Jue and Professor Fan Heping, and it is upon this foundation that my research on moral autonomy continues to build.

2 Wang Yangming stated, “Knowledge is the beginning of action, and action is the completion of knowledge” (Record for Practice, entry 5). This indirectly indicates that although adolescents possess a certain degree of moral knowledge, if they are unable to act, they cannot achieve true understanding—in other words, their knowledge remains incomplete.
